# The SGML Standardization Framework and the Introduction of XML

**DOI:** 10.2196/jmir.2.2.e12

**Published:** 2000-06-30

**Authors:** Walter Fierz, Rolf Grütter

**Affiliations:** ^1^Institute for Clinical Microbiology and ImmunologyUniversity of St. GallenSwitzerland; ^2^Institute for Media and Communications ManagementUniversity of St. GallenSwitzerland

**Keywords:** Standard Generalized Markup Language (SGML), Extensible Markup Language (XML), standardization framework, healthcare, patient record, Architectural Forms, Health Level 7, Swiss HIV Cohort Study (SHCS), clinical study

## Abstract

Extensible Markup Language (XML) is on its way to becoming a global standard for the representation, exchange, and presentation of information on the World Wide Web (WWW). More than that, XML is creating a standardization framework, in terms of an open network of meta-standards and mediators that allows for the definition of further conventions and agreements in specific business domains. Such an approach is particularly needed in the healthcare domain; XML promises to especially suit the particularities of patient records and their lifelong storage, retrieval, and exchange. At a time when change rather than steadiness is becoming the faithful feature of our society, standardization frameworks which support a diversified growth of specifications that are appropriate to the actual needs of the users are becoming more and more important; and efforts should be made to encourage this new attempt at standardization to grow in a fruitful direction. Thus, the introduction of XML reflects a standardization process which is neither exclusively based on an acknowledged standardization authority, nor a pure market standard. Instead, a consortium of companies, academic institutions, and public bodies has agreed on a common recommendation based on an existing standardization framework. The consortium's process of agreeing to a standardization framework will doubtlessly be successful in the case of XML, and it is suggested that it should be considered as a generic model for standardization processes in the future.

## Introduction

Extensible Markup Language (XML) Version 1.0 was endorsed as a Recommendation by the World Wide Web Consortium (W3C) in February 1998 (http://www.w3.org/TR/1998/REC-xml-19980210). W3C is a consortium assembling representatives from many of the leading IT companies, academic institutions, and public bodies [1]. Following W3C's recommendation, all major software companies refocused their development strategies to include XML, and many are now offering products implementing the recommendation. These products range from XML parsers implemented in different programming languages, to sophisticated XML-oriented database applications (for a list of tools that support XML see [2]). XML is also supported by the recent version of Microsoft Internet Explorer and will be supported by the next version of the Netscape browser. The speed of general acceptance and widespread use recalls the introduction of the programming language Java a few years ago and - although public awareness has partly shifted to other issues - imagine where we would be today without it!

The objective of this report is to provide an overview of XML and associated standards. Particular emphasis will be given to its applications in the healthcare sector. This overview will include the history of XML and elucidate the factors enabling the rapid diffusion of XML. In addition, emphasis will be put on the standardization process at the two levels involved: the first includes the acceptance of XML as a syntactical specification; the second considers the agreement upon semantic conventions by specific groups of users, whereby the Document Type Definitions (DTDs) provide the basis for those agreements.

## XML Standardization Framework

### A Standardization Framework

Information technology (IT) standards usually put some constraints on ways that human or computer agents interact, in order to allow collaboration on a common basis. In this report, we would like to concentrate on an additional function of a certain type of standards that might be called meta-standards. Meta-standards specify how other standards can be defined. In principle, one can imagine a whole hierarchy of meta-standards and standards that have their application at different levels of specificity. Corresponding to this kind of standards tree, there are different levels of standard bodies responsible for the specifications of the standards, with the scale reaching from the International Standards Organization (ISO) at the top, via various levels of academic and industry initiatives, ultimately down to the level of two communicating individuals who agree on some mutual way of expressing and handling information objects. An additional type of standard is created to serve the function of specifying ways to translate between different standards, in order to allow the transformation of information objects obeying one standard into objects following a different standard. This type of standard can be called a mediator. Again, there are meta-standards that specify how to define specific mediators.

A system of meta-standards and mediators is what we propose to call a standardization framework. Such a system is able to function in diversified areas of IT, and has the potential of being flexible and adaptable to change. Furthermore, the openness of such a system enables rapid development in growing fields. At a time when change rather than steadiness is becoming the faithful feature of our society, standardization frameworks which support a diversified growth of specifications that are appropriate to the actual needs of the users are becoming more and more important; and efforts should be made to encourage this new attempt at standardization to grow in a fruitful direction. With time, rules might develop that define how such an open system works best. Here, we would like to give an example of a success story of such a standardization framework in the IT area.

### SGML/XML: The Growth of a Standards Tree

The last ten years have seen the tremendous development of a hierarchy of standards and quasi-standards that is based on a common root, the Standard Generalized Markup Language (SGML). Particularly, one application of SGML, Hypertext Markup Language (HTML), has revolutionized the development of the World Wide Web (WWW), the way that electronic documents are interchanged on a global scale. A second revolution is on the horizon: another child of SGML, XML, will profoundly transform the way electronic data will be exchanged. The secret behind this second revolution can be summed up by XML's eponymous attribute, "extensible." While extensibility was already a property of SGML, the improved capabilities of XML have enabled the growth of a family of sub-standards that may be flexibly adapted to the needs of particular domains of users. Additionally, it is conceivable to create ad hoc conventions for data exchange between single individuals who do not belong to a pre-defined group. In this view, SGML/XML has not only revolutionized document and data exchange; perhaps even more importantly, it has moved the vision of standards away from a fixed, centralized, and authoritarian paradigm to a libera,l adaptable system of quasi-standards that simply fulfills the actual needs of communication while adhering to a common framework.

**Figure 1:**
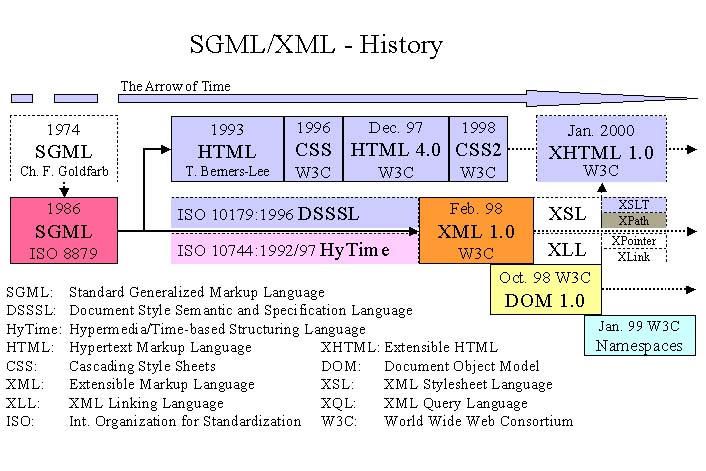
The history of SGML/XML and related standards

To quote Liora Alschuler:

The central problem of information exchange is the tension between: local specialization and global generalization. In other words, "Why can't everyone just agree to do things my way?" is not the right question. The right question is: "How can we impose minimal constraints on local practice yet...ensure that senders and receivers can share meaning where meaning is, indeed, shared? [3]"

In the following sections, the history of the growth and unfolding of the SGML/XML tree of standards will be sketched, and some of its branches will be explained in more detail. Particular reference will be made to its use in the healthcare domain. Figure 1 summarizes the history of SGML/XML and related standards and recommendations.

### SGML (ISO 8879:1986)

The general idea behind SGML is to provide rules that are able to define what the content of a document is, rather then what the document looks like. The separation of the content and structure of a text from the style information used for its rendering in an output device is an important precondition for the document not only to be accessible with heterogeneous software systems, but also to survive in an environment of constantly changing software tools. Inadvertently, this concentration of SGML on the content and structure of a text has made it possible to use SGML not only for documents, but also for data in general, a development that is just beginning now. Furthermore, the origin of SGML in the publishing area has led to a syntax that is not only machine-interpretable but at least in principle also human-interpretable. This, again, is not unimportant when considering the longevity of certain documents.

The technique that is used by SGML is Markup. Simply speaking, a piece of text or data is enclosed between two tags that "mark it up" as something defined. Such markup allows machine agents to to manipulate the content and to assign a semantic meaning to the piece of data. SGML provides the syntax to define these tags and the rules that govern the use of the markup tags. These definitions are placed either in the prolog of the document or in a separate document called a Document Type Definition (DTD). The overall structure defined by the rules of SGML is that of a tree of elements with one single root element. Each element-type is defined in the DTD by its tag-name and its attributes. The rules given in the DTD govern the possible occurrences of the elements in the tree, and the possible attribute values. Apart from elements and their attributes, the DTD might also define entities. These are character strings that stand for something else, such as longer strings, special characters, groups of elements, or items stored in an external file like graphics or text fragments.

Historically, SGML emerged in the late 60's in the publishing industry, which had a need to establish generic typesetting codes that would allow the text of a document to be manipulated in different text processing systems. One of these approaches was the Generalized Markup Language developed by Goldfarb, Mosher, and Lorie at IBM. A key concept was the DTD, which allowed the construction of markup rules for specific applications and the use of parsers to validate the syntax of the document markup for its appropriateness to the particular application. Another impetus came from a committee of the Graphic Communications Association (GCA) that created GenCode to standardize typesetting codes. With a collaborative international effort in the 70's under the technical leadership of Charles F. Goldfarb, SGML was developed into an international standard which was adopted by the International Organization of Standards as ISO 8879 in 1986. It has been proven since to be a robust, stable standard that has led to the introduction of manifold applications in the form of further standards, quasi-standards, and academic as well as industry initiatives.

Since only a very short overview over the basic principles of SGML can be given here, we refer for further details to some books dedicated to describing SGML and its applications [4-6].

### SGML Applications

The openness and extensibility of SGML stems from the basic concept that the DTD is constructed by the user, or group of users, of the standard. In this sense, SGML is a syntax that allows the definition of further standards. Each DTD defines the particular markup structure that is allowed in a document, and builds a basis by which semantic meaning is conferred on the marked-up content.

#### Industry-Standard DTDs

Some of the major applications of SGML, in form of DTDs, have been created by industry groups to facilitate document processing and exchange. Table 1 gives a short overview (compiled from [7]).

**Table 1:** Industry-Standard DTDs

Name	Description	URL / Public Identifier
ISO 12083:1993	International standard defining three DTDs: - books - serial publications - individual articles	ISO 12083:1993//DTD Book//EN ISO 12083:1993//DTD Serial//EN ISO 12083:1993//DTD Article//EN
DocBook	Consortium standard (OASIS) for publishing books with additional features for computer and software documentation	-//OASIS//DTD DocBook V3.1//EN [8]
Text-Encoding Initiative (TEI)	Book-oriented DTD for research-oriented applications. Subset for simpler applications: TEI-Lite	-//TEI//DTD TEI Lite 1.0//EN [9]
MIL-STD-38784 CALS	Book-oriented DTD for technical documents. Known for its table model (CALS) that became a de facto standard for tables.	-//USA-DOD//DTD MIL-STD-38784 AMEND1//EN [10]

Despite its sound and open design, SGML was for some time only used in publishing, mainly by large-scale government and industry enterprises that had to deal with complex documents. It was only when a relatively simple DTD of SGML was combined with a linking mechanism and coded into an Internet-based hypertext application that SGML got into widespread use in form of Hypertext Markup Language (HTML).

#### HTML and the World Wide Web

As the global public gained access to the former ARPAnet, the Internet evolved; several information services evolved along with it, with the multimedia, interactive WWW gaining the favor of users. In accordance with the hypertext paradigm [11], which refers to a non-sequential linking of information objects, the idea was (and still is) to author and exchange hyper-linked documents. For the implementation of this idea, the SGML standardization framework proved to be an excellent foundation.

The birth of HTML took place 1989 at CERN, where it was developed by Tim Berners-Lee to share hyperlinked text documents within the CERN European Nuclear Research Facility. The new SGML application was made available openly on the Internet, and it soon became very popular and rapidly developed into the WWW as it is well-known and used all over the world today. To be precise, HTML was, at its beginning, not a strictly-conforming SGML application; its development was mainly driven by numerous programmers on the Internet and by the competing browser vendors. In this sense, HTML developed rather like a de facto standard. Fortunately, the World Wide Web Consortium (W3C), the Web's standards body, managed to get HTML under control and produced DTDs for formal description of each new version of HTML. The first SGML-compliant version was HTML 2.0.

Another departure of HTML from the principals of SGML was perhaps more serious. HTML concentrated mainly on the layout of a document rather then on its content. This development went so far that element tags and attributes were used to directly define formatting instructions. Thus the basic SGML concept of separating content and structure from format was violated. Efforts were made in 1996 by the W3C to halt this development with the introduction of Cascading Style Sheets (CSS). Style sheets have from the beginning been used with SGML to define the formatting of structured text (see section DSSSL). The new versions of HTML browsers are now able to deal with CSS.

HTML is now in its fourth version, which was released by the W3C in December 1997 [12] . The second version of CSS has passed as a W3C Recommendation in May 1998 [13]. Further development of HTML will likely go into the direction of XML (see below), i.e. it has been reformulated as an application of XML and is called XHTML (see below).

#### HyTime (ISO/IEC 10744:1997)

Hypermedia/Time-based Structuring Language (HyTime) [14] is an SGML application that specifies a way in which logically connected or time-related information can be described within the framework of SGML. One of its origins is rooted in the attempt to create a language to describe music. However, it can be used for any type of linking between related or time-based information objects.

The main goal of HyTime is to provide rules for linking information objects and scheduling within finite coordinate spaces. In different HyTime modules, methods for addressing locations and methods for hyperlinking between those addresses are defined. Other HyTime modules contain facilities for scheduling and rendition of events within event schedules. Information about HyTime can be found at the site of the HyTime Users' Group [15].

The design principle of HyTime is a meta-DTD, a kind of template called *architectural form* or *enabling architecture*. HyTime lets the user of the standard define his or her own DTDs and relate them to the architectural form with a specific *Hytime* attribute. HyTime-aware applications can recognize these attributes and apply to the corresponding elements any specific processing designed for the HyTime template. Annex A.3 to the second edition of the HyTime standard (ISO/IEC 10744:1997) contains the definition of architectural forms: "Architectural Form Definition Requirements (AFRD)" [16], whereas Annex C standardizes the meta-DTD formalism used for architectures. A general description of SGML architectures by Steven R. Newcomb can be found at [17].

##### Architectural Forms

HyTime itself is the first, pioneering SGML architecture, but other architectures can be constructed and be used for specific applications. Architectural forms are particularly useful when a user would like to create a DTD that adheres to an industry-standard DTD, but would still like to use his or her own customized tag names.

Since each DTD can serve, in principle, as a meta-DTD, architectural forms can be used to build up whole hierarchies of DTDs or standards. The property of such hierarchical DTDs to inherit the constructs of the meta-DTD a level above makes architectures very similar to classes in object-oriented systems. In fact, DTDs can even inherit from multiple enabling architectures.

###### The Kona Proposal for a Patient Record Architecture (PRA), an Enabling Architecture in Healthcare

Currently, attempts are being made to construct architectural forms for the healthcare sector by the Kona Editorial Group [18]. A three level architecture is being proposed as a Patient Record Architecture (PRA) for the exchange of clinical documents. The information model for this architecture is the HL7 Reference Information Model (RIM [19]). The least granular level of the architecture (Level 1) only encodes the header information, but leaves the rest of the document in plain text form. Level 2 structures the document into sections to allow minimal processing, whereas Level 3 will be consistent in granularity with the RIM. The idea of using an architectural form for structuring patient records is very promising, since it will hopefully allow the system to cope with the wide variety of local needs for specific structures and still enable data exchange under the umbrella of a common standard.

##### Groves and Property Sets

The basic idea underlying groves is that SGML is merely a syntax for some underlying data model: the tree or, to be precise, a collection of trees. The grove provides a language for describing SGML's abstract data model. The grove is an abstract meta-"data-model" containing nodes with properties. The grove model for SGML itself or an SGML application like HyTime is specified with properties collected in *property sets*. The grove paradigm is defined in Annex A.4 to the HyTime standard (ISO/IEC 10744:1997) in the "Property Sets Definition Requirements (PSDR)" [20]. An introduction to groves and property sets by Paul Prescod can be found at [65].

#### Topic Navigation Maps (ISO/IEC 13250)

Topic Maps are an application of HyTime that uses its powerful model of universal addressing and independent linking. This international standard defines a notation for representing information about the structure of resources used to define topics, and the relationships between topics. Filters can be used either to include or to exclude information. A set of interrelated documents that employs the notation defined by this standard is called a Topic Navigation Map (TNM). Topic Maps are expressed as a set of architectural forms. As with HyTime, the TNM standard does not require a particular DTD to be used. It is an architecture that serves as template for adding attributes to elements in any DTD that can fit a specific environment [21]. There are no tools available at present to handle Topic Maps, but it is foreseeable that Topic Maps might play a significant role in the future as a syntax used to express semantic relations. Hopefully, such approaches will also be undertaken in the medical area where the problem of sementic diversity is considerable.

#### Document Style Semantics and Specification Language (DSSSL, ISO/IEC 10179:1996)

As discussed above, one of the basic principles of SGML is the separation of content and structure of a document from the style information used for its rendering in an output device. Complimentary to SGML, a standard was developed that defines rules for processing SGML documents: the Document Style Semantics and Specification Language (DSSSL). It contains three parts, a Style Language for formatting, a Transformation Language for transforming, and a Query Language for extracting data. DSSSL is closely related to SGML but is not an application of SGML. Information about DSSSL can be found at a site maintained by James Clark, the author of Jade, a freely available DSSSL-engine [22].

### Back to the roots: XML - a subset of SGML

The success of HTML with the explosive expansion of the WWW is partly due to its relatively simple specifications, which allowed its rapid adoption by programmers and users on the Internet. However, due to its fixed DTD, each introduction of new element types required a new version of HTML, which made it somewhat inflexible. Indeed, software companies tended to extend HTML beyond its specifications, endangering its function as a quasi-standard. In 1996, a working group was formed under the auspices of the W3C with the goal of solving the problem of extensibility. Remembering the roots of HTML, it considered adapting SGML with its open paradigm - which was so far not successful on the Web - to the needs of the Web community. The result of this endeavor was the creation of a subset of SGML, the eXtensible Markup Language (XML), that was easier for programmers to handle; this consequently encouraged widespread adoption.

XML 1.0 was accepted as a recommendation by the W3C in February 1998 and it has enjoyed an acceptance by the software industry that goes beyond all expectations. The specifications of XML have been annotated by one of its creators, Tim Bray, and can be found at [23]. For a more detailed introduction to XML see [24,25].

#### SGML/XML Applications

Even before its final specifications were accepted in 1998, several applications of XML were created by the community of users: Chemical Markup Language (CML) to manage chemical information [26]; or Mathematical Markup Language (MathML), for describing mathematical notation [27], which was accepted as a W3C Recommendation in April 1998 (revised July 1999).

Another early application of XML is the Resource Description Framework (RDF) for the processing of metadata and the provision of interoperability between applications that exchange machine-interpretable information on the Web [28]. It was recommended by W3C in February 1999.

An excellent compilation of SGML/XML applications is maintained by Robin Cover at OASIS's SGML/XML Web page under the headings:

- General Applications [29];
- Academic Applications [30];
- Industry Applications [31].

As an example we will discuss some applications in the healthcare sector.

#### XML in Healthcare

Healthcare is to a large extent an information-processing activity. Data about the patient's physical condition are collected by the treating physician using various diagnostic techniques, and evaluated within the framework of his or her medical knowledge to reach the appropriate decision for therapeutic measures or further diagnostic procedures. If this information processing path is to be effectively enhanced by electronic decision support systems, it is inevitable that data are to be structured at some time point, ideally at the very moment of data collection.

For this structuring to be useful, however, it requires a standard syntax and terminology that is used by all participitating healthcare providers. The lack of such a commonly agreed-upon electronic language has so far been a major impediment for rapid development in this field. EDI (Electronic Data Interchange) standards like HL7 or UN/EDIFACT have found a certain application, but mainly in the administrative and financial areas of healthcare. For the first time, XML provides a concept and technology that promises to provide a flexible, open, and standardized solution to the problems of structuring, storing, and exchanging patient data. The independence of XML from particular software vendors, its self-describing nature, and not least, the fact that XML can be read by human beings as well as by computer programs, makes XML particularly suited to storing and handling documents and data over a long period of time, as it is needed for patient records.

##### HL7 SGML/XML Special Interest Group and the Task Force XML of CEN TC251

Health Level 7 (HL7) was founded in 1987 to develop standards for the electronic interchange of clinical, financial, and administrative information among independent healthcare oriented computer systems; e.g., hospital information systems, clinical laboratory systems, enterprise systems, and pharmacy systems. In August 1996, the HL7 Technical Steering Committee authorized the creation of an SGML Special Interest Group as part of a larger initiative to integrate SGML into medical informatics standards. "HCML" is a proposed abbreviation for the evolving markup language: "Health Care Markup Language" [32]. In December 1998, a draft document was produced as a proposal for using "XML as an Interchange Format for HL7 V2.3 Messages" [33]. In addition, an XML-based Patient Record Architecture (PRA) is being completed at Level One (see above).

In Europe, an XML task force has been established by CEN/TC 251 to investigate various aspects of using XML syntax for health messages and documents [34].

**Figure 2:**
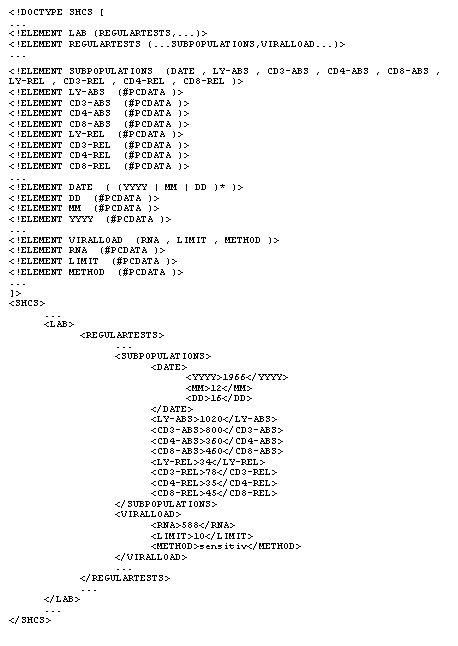
The SHCS Web document type definition and XML document (excerpts)

##### SHCS Web: The use of XML in multi-center clinical studies

The extensibility of XML makes it particularly useful for the definition of syntactic rules and semantic conventions for communication within a domain of users with a specific task. We are using XML as WWW-based middleware in order to establish communication in a distributed and heterogeneous systems environment of a clinical multi-center study, the Swiss HIV Cohort Study (SHCS) [35].

Unlike HTML, XML allows for the explicit declaration of element types and representation of document structure in the DTD (see Figure 2). As stated for SGML, the names of the tags (i.e. the semantics of the terminal syntactical constructs) have to be fixed by convention. In our case, we use selected concepts as part of a domain-specific language (i. e. clinical immunology) as tag names (e.g. <viralload>). This approach yields the advantage that the XML documents can be interpreted by both machines and humans, thereby providing an excellent basis for cooperation among human and artificial agents.

The DTD for the SHCS Web application was set up using the preexisting paper-based study form as a template. So in essence, the paper study form was transformed into a structured electronic study form (ESF). One of the design problems in defining the DTD for the ESF was deciding in which cases data should be represented as attribute values and in which cases as element contents. As we began to work with XML even before February 1998, we faced the lack of others' experience with the standard. Thus, we decided in the beginning to pursue an attribute-oriented approach. In the meantime, the situation has changed. Today, a considerable number of XML applications exist, and practices, such as the previously mentioned question of how to represent contents, are being established. Thus, it appears that data are usually represented as element contents whereas attributes are preferably used for the representation of meta-information and references.

This change of DTD confronted us with the problem that we had to find a way to transform the old XML files to ones that followed the new DTD. We reached a solution by making use of the newly developped XSLT specification (see below) that provides an elegant way to deal with such problems. This course of events gives a perfect example of the flexibility of XML and the possibility of mediating between two different ad hoc standards of XML applications.

For more information about SHCS Web see Fierz & Grütter [36-38].

##### Other Examples of Healthcare Applications

The idea of using a generalized language for platform-independent structured reporting in healthcare had already been realized before the time of XML. A Data-entry and Reporting Markup Language (DRML), an SGML application, had been formulated by Kahn [39]. In Wales, a NHS project with the aim of structuring patient records had originally been based on SGML, but has moved now to XML. Over 250,000 patient records from the Orthopaedic Hospital Trust in Oswestry have already been translated into XML [40]. The main advantages as seen by the initiators of the project lie in the possiblity of querying the patient's XML database in a much more efficient way than would have been possible with a free text search.

#### Doing Business with XML

##### XML/EDI

According to a founding member of the XML/EDI Group initiative [41], the overall idea of XML/EDI is to add enough intelligence to the documents so that they become the framework for electronic commerce. Thereby, XML/EDI should define a standard for encoding the presentation characteristics, structure, and behavior of data that supports business transactions. Not only data should be delivered, but also information and the processing logic that is required to make sense of it. (Information is distinguished from data by its high-level semantics, referring to real-world objects, whereas the low-level semantics of data, i. e. the elementary data types, indicate only whether a given bit stream should be interpreted as CHAR, INTEGER etc.).

**Figure 3:**
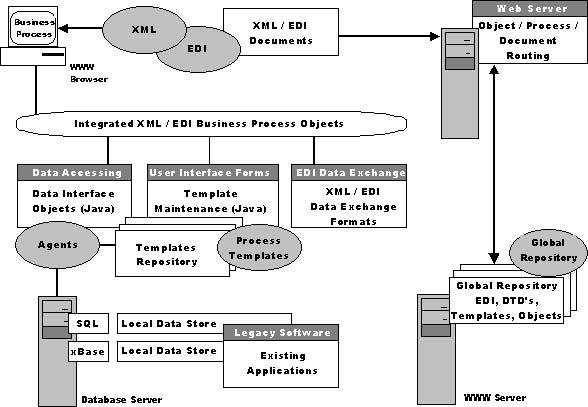
An integrated XML/EDI Internet-based system (Webber, 1998, reproduction with permission from the author)

Webber identifies five components of an Integrated XML/EDI Internet-based System (see Figure 3):

- XML: The XML container transports the other components across the network. Thereby, XML tokens replace or supplement existing EDI segment identifiers. The system further takes advantage of the rich capabilities and transport layers of the Web and the Internet.
- EDI: Old EDI is called the grandfather of the current electronic commerce. Implementations include ANSI X.12 in the United States and UN/EDIFACT in Europe. XML/EDI provides 100% backward compatibility with existing EDI transactions, thereby preserving investments in existing EDI systems and knowledge.
- Templates: Process Templates enable processing of transactions (whereas Document Type Definitions (DTDs) enable transaction interoperability by defining the structure and content). They are globally referenced or travel along inside XML. Process Templates resemble traditional process control language syntax.
- Agents: Software Agents interpret the Process Templates to perform the work needed. They further interact with the EDI transaction data definitions and the users' business applications to create new templates for each new specific task.
- Repositories: Global Internet Repositories provide the semantic foundation for global business transactions. They allow for automatic lookups of the meaning and definition of the EDI elements. Global Internet Repositories are supplemented by adding DTDs and Process Templates. The XML/EDI group has compiled a "White Paper on XML Global Repositories for XML/EDI" [42].

The real leverage of XML/EDI, when compared with traditional EDI, derives from the possibility of Partner A to communicate with Partner B based on the data format of their local business applications, instead of the necessity for both to conform to a standard. The system creates, with some manual help, a template describing the local record structures and field definitions. This template is added to the XML container and sent to Partner B along with the data. There it allows the receiving Partner B to create a second template defining the mappings of the data from Partner A onto the database system of his or her own business application as well as the rules that correspond to a set of necessary data transformations. During these processes, partner B is assisted by software agents. The first time, this mapping must be done manually. Once the template B is generated, data exchange from A to B can be automated. In other words, XML/EDI allows the creation of an ad hoc communication standard between A and B.

Currently, the XML/EDI framework is based on "Guidelines for using XML for Electronic Data Interchange" developed by the XML/EDI group [43]. XML/EDI pilot projects are under way for X.12-based data exchange [44], as well as for the European part of EDI, i.e., EDIFACT [45].

##### XMI

XML Metadata Interchange Format (XMI) is a new open industry standard that combines the benefits of the Web-based XML standard for defining, validating, and sharing document formats on the web with the benefits of the object-oriented Unified Modeling Language (UML). It provides application developers with a common language for specifying, visualizing, constructing, and documenting distributed objects and business models.

The objective of XMI is to allow the exchange of objects from the Object Management Group's (OMG) Object Analysis and Design Facility. These objects are more commonly described as UML and MOF (Meta Objects Facility) [46].

#### XML-related Specifications

##### Document Object Model (DOM)

###### Version 1.0, W3C Recommendation 1 October, 1998

This specification defines the Document Object Model Level 1, a platform- and language-neutral interface that allows programs and scripts to dynamically access and update the content, structure and style of documents. The Document Object Model provides a standard set of objects for representing HTML and XML documents, a standard model of how these objects can be combined, and a standard interface for accessing and manipulating them. Vendors can support the DOM as an interface to their proprietary data structures and APIs, and content authors can write to the standard DOM interfaces rather than product-specific APIs, thus increasing interoperability on the Web [47].

An extension of the DOM Level 1 was being recommended by W3C in October 1999 as DOM Level 2 [48].

##### Namespaces in XML

To use DTDs and XML documents in a modular way, it is often desirable to be able to combine different DTDs within one XML document or to integrate XML documents that use different DTDs. However, since different DTDs might not recognize each other, they might use the same element names for different semantic entities and different element names for the same semantic entities, thereby inducing name collisions and semantic heterogeneity. In a W3C Recommendation of January 1999, rules for namespaces are described to solve this problem. XML namespaces provide a simple method for qualifying element and attribute names used in XML documents by associating them with namespaces identified by URI references [49].

### Current and Future Developments

XML is just at its beginning. Its inclusion in a standardization framework paves the way for the development of further standards. Some of them are foreseeable and actually already under construction (see below). Others, however, might not yet be accessible to our imagination.

#### XSL

Extensible Stylesheet Language (XSL), an application of XML, is a language for expressing rendering information. It consists of a language for transforming XML documents, and an XML vocabulary for specifying formatting semantics. Thus XSL, like DSSSL that guided its conception, goes beyond merely specifying a syntax for defining style information. In a broader view, it is a transforming language that allows the conversion of documents obeying one DTD into documents referring to another DTD. It also contains elements for querying, what makes it a basis whereupon a query language can be built.

In March 2000, the W3C made the latest working draft of XSL 1.0 available [50]. In November 1999, the transforming part of XSL was issued as separate Recommendation called XSLT [51], and XPath designated as a language for addressing parts of an XML document, designed to be used by both XSLT and Xpointer (see below) [52]. A way to associate Style Sheets with XML documents has been specified and recommended by the W3C in June 1999 [53].

#### XQL

Beyond the original goal of SGML to standardize interchange of documents, XML will play an important role for interchange of any kind of data on the web. In fact, database tools that serve the XML standard are already on the market. The object-oriented paradigm is particularly suited for this purpose, but more importantly, a whole new approach in database design has already led to the first native XML database that preserves the original XML structure. However, a new XML-oriented query language is needed for searching, filtering, and retrieving data. In December 1998, the W3C convened a workshop on query languages called QL'98 [54]. A total of 66 position papers [55] from about 30 companies and 25 academic institutions and research facilities show the wide interest in the query issue. An extensive report on QL'98 has been compiled by Lisa Rein [56].

#### XLL

The success of HTML is in large part grounded on its simple linking mechanism that allowed programmers to turn the hypertext paradigm into worldwide reality. Preserving the linking functionality is therefore very important for XML. However, the linking mechanism provided by HTML again is somewhat limited, leading for example to the well-known problem of lost links on the WWW. The key concepts for an extended linking functionality are defined in the HyTime standard (see above). One basic idea is to separate the linking part from the addressing part to ease the maintenance of links. So, eXtensible Linking Language (XLL) as a broad term for XML hyperlinking (linking and addressing) has two major components: A linking language (XLink) and an addressing language (XPointer). XLink and Xpointer are currently (as of July 2000) candidate recommendations of the W3C [57,58].

#### XML Schema

The syntax of the Document Type Definition (DTD) is somewhat limited in its capability to express constraints on specific classes of documents. So, it does not provide, for example, a mechanism to describe primitive datatypes or default values for element contents. In February 1999, the W3C has issued requirements for a schema language [59] that uses XML syntax. Such a construct should allow, among other things, the import and export of datatypes from and to database systems, and the creation of user-defined datatypes. Two proposals for such a language have been submitted to the W3C: XML-Data [60], and Document Definition Markup Language (DDML) [61]. In April 2000, the W3C has issued two working drafts: XML Schema Part 1 for Structures [62], and XML Schema Part 2 for Datatypes [63].

#### XHTML

In January 2000 the W3C issued a recommendation for a reformulation of HTML 4.0 in XML 1.0: the Extensible HyperText Markup Language (XHTM™ 1.0). This specification defines HTML 4.0 as an XML application. The semantics of the elements and their attributes are defined in the W3C Recommendation for HTML 4.0. These semantics provide the foundation for future extensibility of XHTML. Compatibility with existing HTML user agents is possible by following a small set of guidelines [64]..

## Conclusion

The described introduction of XML as a syntactical specification reflects a standardization process which is neither exclusively based on a binding decision of an acknowledged standardization authority (such as ISO) nor a pure market standard (or de facto standard) which is often a result of some monopolistic power (e.g., the operating system Windows). Instead, based on a standardization framework, a consortium of companies, academic institutions, and public bodies has agreed on a common recommendation. This story of the evolution of a standardization framework doubtlessly will end successfully in the case of XML, and we suggest that it should be considered as a generic model for standardization processes in the future.

The healthcare area is especially in need of such a standardization process, because of two main reasons: First, patient care has become more and more a process involving multiple providers, and rapid information exchange between the providers is pivotal not only to the patient's health, but also to the economic viability of healthcare. Second, the handling of patient data is a lifelong process that should not be affected by the ripples of vendor-specific software specifications.

With respect to the handling of semantic specifications, the old controversy of global standards that may be implemented as distributed and uniquely referable repositories versus local conventions has been adopted to the subject of semantic DTD schema specification. As mentioned, we think that the decision must not be in favor of either the global or local approaches, but should aim at their integration. There are actually two practical solutions to the problem of integration of local conventions into global standards. The first solution is a hierarchical approach which aims at aggregating local schemas into a general global schema. The second solution is a peer approach which aims at mediating between different schemas. The above-mentioned XML Stylesheet Language (XSL), particularly its transformations specification (XSLT), supports the second approach in that it allows a transformation between documents referring to different DTDs. Alternately, architectures allow for a mapping of different DTDs onto an aggregated one. However, as aggregation is always an abstraction from detailed information, the mapping of DTDs based on architectures can bring along a loss of information.

**Figure 4:**
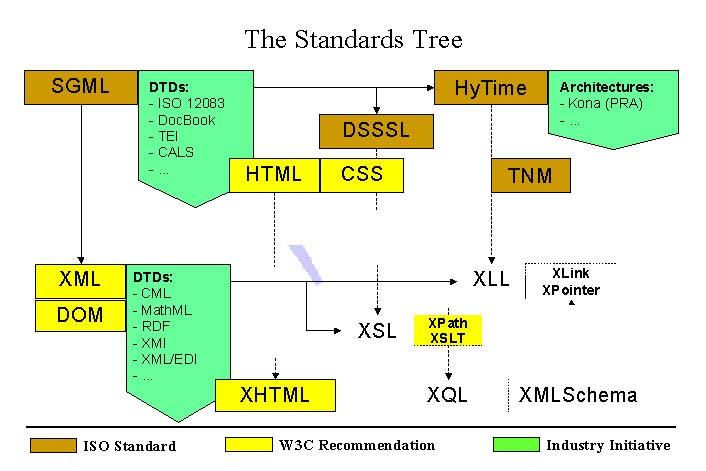
The logical connections between SGML/XML and related standards (abbreviations explained in the text)

The possibility of constructing DTDs for transformation or architectural aggregation is an illustration of what we like to call a standardization framework (see Figure 4). But the story goes further: not only are SGML/XML meta-standards that have led to a rapid growth of applications in the form of new standards and meta-standards within just one year; but the process continues, particularly in the business area, and likewise in the healthcare domain. DTDs and templates will be established for particular business domains with an ever-increasing granularity of specification. It leads to a fractal behavior of the standardization system enabling a diversified growth within a common framework. This paradigm is close to nature.
